# Performance of deep learning synthetic CTs for MR‐only brain radiation therapy

**DOI:** 10.1002/acm2.13139

**Published:** 2021-01-07

**Authors:** Xiaoning Liu, Hajar Emami, Siamak P. Nejad‐Davarani, Eric Morris, Lonni Schultz, Ming Dong, Carri K. Glide‐Hurst

**Affiliations:** ^1^ Department of Medical Physics Memorial Sloan Kettering Cancer Center Middletown NJ USA; ^2^ Department of Computer Science Wayne State University Detroit MI USA; ^3^ Department of Radiation Oncology University of Michigan Ann Arbor MI USA; ^4^ Department of Radiation Oncology University of California—Los Angeles Los Angeles CA USA; ^5^ Department of Public Health Sciences Henry Ford Health System Detroit MI USA; ^6^ Department of Human Oncology School of Medicine and Public Heath University of Wisconsin – Madison Madison WI USA

**Keywords:** Deep Learning, General Adversarial Network, Image Guided Radiation Therapy, Synthetic CT

## Abstract

**Purpose:**

To evaluate the dosimetric and image‐guided radiation therapy (IGRT) performance of a novel generative adversarial network (GAN) generated synthetic CT (synCT) in the brain and compare its performance for clinical use including conventional brain radiotherapy, cranial stereotactic radiosurgery (SRS), planar, and volumetric IGRT.

**Methods and Materials:**

SynCT images for 12 brain cancer patients (6 SRS, 6 conventional) were generated from T1‐weighted postgadolinium magnetic resonance (MR) images by applying a GAN model with a residual network (ResNet) generator and a convolutional neural network (CNN) with 5 convolutional layers as the discriminator that classified input images as real or synthetic. Following rigid registration, clinical structures and treatment plans derived from simulation CT (simCT) images were transferred to synCTs. Dose was recalculated for 15 simCT/synCT plan pairs using fixed monitor units. Two‐dimensional (2D) gamma analysis (2%/2 mm, 1%/1 mm) was performed to compare dose distributions at isocenter. Dose–volume histogram (DVH) metrics (D_95%_, D_99%_, D_0.2cc,_ and D_0.035cc_) were assessed for the targets and organ at risks (OARs). IGRT performance was evaluated via volumetric registration between cone beam CT (CBCT) to synCT/simCT and planar registration between KV images to synCT/simCT digital reconstructed radiographs (DRRs).

**Results:**

Average gamma passing rates at 1%/1mm and 2%/2mm were 99.0 ± 1.5% and 99.9 ± 0.2%, respectively. Excellent agreement in DVH metrics was observed (mean difference ≤0.10 ± 0.04 Gy for targets, 0.13 ± 0.04 Gy for OARs). The population averaged mean difference in CBCT‐synCT registrations were <0.2 mm and 0.1 degree different from simCT‐based registrations. The mean difference between kV‐synCT DRR and kV‐simCT DRR registrations was <0.5 mm with no statistically significant differences observed (*P* > 0.05). An outlier with a large resection cavity exhibited the worst‐case scenario.

**Conclusion:**

Brain GAN synCTs demonstrated excellent performance for dosimetric and IGRT endpoints, offering potential use in high precision brain cancer therapy.

## 1 Introduction

Magnetic resonance imaging (MRI) provides superior soft tissue contrast than computed tomography (CT), the gold standard image modality used for treatment planning in radiotherapy. The incorporation of MRI as an adjunct to CT significantly reduces inter/intraobserver variations in structure delineation.^1^ As a complimentary modality, the MRI is registered to simulation CT (simCT) to transfer MRI delineated structures for treatment planning. However, this multimodality registration may introduce up to ~ 2 mm of systematic error in the head region.^2^, ^3^, ^4^ In an effort to eliminate multimodality image registration uncertainty and improve clinical efficiency, MR‐only treatment planning has emerged as a viable treatment option for many disease sites.^5^, ^6^, ^7^

Yet, implementing MR‐only treatment planning presents several challenges including that MRI does not provide electron density information required for accurate dose calculation. In the brain, several synthetic CT (synCT) generation methods from MRI data have been developed including bulk density assignments, atlas‐based, voxel‐based, and machine learning‐based methods.^8^, ^9^, ^10^, ^11^, ^12^, ^13^, ^14^ Recently, deep learning has achieved superior accuracy in synCT generation than other approaches.^8^, ^9^, ^10^, ^11^, ^12^ Wang *et al*.^15^ reported that synCT generated from a path‐based random forest method achieved less than 0.6% dose difference in target DVH metrics and a 99% average gamma passing rate (3%/3mm) in brain stereotactic radiosurgery (SRS) treatments. Kazemifar *et al*.^16^ assessed the dosimetric accuracy of generative adversarial networks (GANs) generated synCT in brain radiotherapy and found <1% dose difference in dose–volume histogram (DVH) endpoints. Despite the existing studies on dosimetric performance, very few studies have assessed the performance of synCT for image‐guided radiation therapy (IGRT). Price *et al*.^17^ and Morris *et al*.^18^ found that synCTs generated using voxel‐based weighted summation achieved similar performance for whole and partial brain IGRT, respectively. However, the synCT method employed required multiple MR datasets to generate synCT images that may have introduced other potential coregistration errors and did not implement deep learning. We recently developed and validated a GAN model that generates brain synCTs from a single MRI input in ~6 s, yielding excellent agreement to the corresponding CT.^12^ This work aims to further evaluate the dosimetric and IGRT performance of GAN generated synCT (GAN‐synCT) in the brain and compare its performance for clinical use including conventional brain radiotherapy, cranial SRS, planar IGRT, and volumetric IGRT.

## 2 METHODS

### 2.1 Data acquisition

A cohort of 12 brain cancer patients treated on Edge, Novalis TX, or TrueBeam linear accelerator platforms (Varian Medical Systems, Palo Alto, CA, USA) were retrospectively evaluated as part of an Institutional review board approved study. Six patients underwent cranial SRS and six underwent conventional brain radiotherapy (with more than five fractions), among which three patients had a boost treatment. Eight patients underwent surgical resection before presenting for radiation therapy. All CT simulations were performed on a Brilliance Big Bore CT scanner (Philips Health Care, Cleveland, OH) with 120 kVp. Per our clinical practice, SRS patients were acquired with a high resolution CT protocol (in‐plane resolution of 0.88 × 0.88 mm^2^, 1 mm slice thickness) while conventionally fractionated brain cases were acquired with an in‐plane resolution of 1.17 × 1.17 mm^2^, 2 mm slice thickness. Patients were immobilized using a thermoplastic head mask during CT simulation, onboard imaging acquisition, and treatments. MR scans were acquired with a 1.0T Panorama High Field Open MR Simulator (Philips Medical Systems, Best, the Netherlands) without any immobilization devices in order to accommodate the eight‐channel head coil. Postgadolinium T1‐weighted images were acquired for each patient with a voxel size of 0.90 × 0.90 × 1.25 mm^3^. On‐board cone beam CT (CBCT) and kilovoltage (kV) planar images were acquired on three different linear accelerators: Edge (nine patients), Novalis TX (two patients), and TrueBeam (one patient) with CBCT slice thickness ranging from 1 to 2.5 mm (0.5 to 0.65 mm pixel size). On‐board kV images were acquired with pixel size of 0.2 × 0.2 mm^2^ and were exported along with digitally reconstructed radiographs (DRRs) from the Eclipse® Treatment Planning System (TPS) (Varian Medical Systems, Palo Alto, CA, USA) using an integrated DICOM filter (Image Browser V15.5) for subsequent registration.

### 2.2 Synthetic CT Generation and Preprocessing

The synCT images were generated using a previously developed GAN deep learning model.^12^ The GAN model trains two competing networks simultaneously: (a) an encoder–decoder architecture called the generator, which tries to generate the synCTs from the input MR images (b) and a discriminator which classifies the generated synCTs as real or synthetic.^19^ The generator’s architecture includes nine residual blocks, where the discriminator is a CNN with five convolutional layers. As outlined in detail in the original developmental work, the GAN model was validated using a fivefold cross‐validation technique. A detailed comparison of GAN synCT and simplified CNN highlighted that our GAN reduced the mean absolute error and better preserved details than CNN.^12^

To ensure equivalent dosimetric and IGRT comparisons were conducted between the simCT reference and synCT, all synCTs were sampled to the CT simulation grid resolution for each patient case for final analysis. All synCT images were then rigidly registered to the corresponding simCT images using Statistical Parametric Mapping software^20^ (SPM12, Functional Imaging Laboratory, The Wellcome Trust Centre for NeuroImaging, University College London). The coregistered synCT images were converted into DICOM and imported into the Eclipse TPS using an in‐house MATLAB (Mathworks, Natick, MA) code. The clinical structures delineated on simCT images were transferred to the coregistered synCT images. To ensure the same dose calculation volumes between each simCT/synCT plan pair, the external body (i.e., structure used to define dose calculation volume) was defined by the synCT dataset.

### 2.3 Treatment planning

A total of 15 clinically treated treatment plans (12 primary plans and 3 boost plans) generated on simCT data using either volumetric modulated arc therapy (VMAT, n = 13) or dynamic conformal arc (DCA, n = 2) were copied onto synCT images. Dose was recalculated for both simCT (with new external body boundary) and synCT plans with fixed monitor units and the same dose calculation grid volumes using the Eclipse Anisotropic Analytical Algorithm (AAA, v.11).

### 2.4 Dosimetric performance evaluation

Dose distributions calculated on synCT images were compared against the simCT dose distributions via three methods: (a) point dose discrepancy in terms of mean error (ME) with standard deviation (SD) evaluated for clinical DVH metrics, (b) plan quality change as indicated by gradient index (GI) and conformity index (CI), and (c) 2D gamma analysis^21^ conducted in the axial plane evaluated at the isocenter location for the synCT and simCT based plans in a manner similar to our standard clinical evaluation.

The evaluated DVH metrics were defined in Quantitative Analyses of Normal Tissue Effects in the Clinic (QUANTEC)^22^ and AAPM Task Group No. 101 Report,^23^ including D_99%_ and D_95%_ for the target, D_0.02cc_ for optic pathways, D_0.05cc_ for brainstem, and D_0.035cc_ for both target and organs at risk (OARs). D_99%_ and D_95%_ represent the doses delivered to 99% and 95% of the planning target volume (PTV), and D_0.035cc_, as a representation of maximum dose, is the dose to 0.035cc of a structure’s volume. GI, defined as the ratio of the volume of half the prescription isodose to the volume of the prescription isodose, describes how fast the dose falls off outside of the target.^24^ CI is the ratio of 100% isodose volume to the volume of the PTV, indicating how well the prescription dose conformed to the target.^25^

Gamma analysis at 1%/1 mm and 2%/2 mm (dose difference/distance to agreement) was conducted using a low‐dose threshold of 10% of the maximum dose in simCT plan using in‐house software. For cases with tumor volumes >100cc, a 30 × 30 cm^2^ dose plane was used to evaluate the global dose distribution. For the remaining cases with small tumor volumes, a 15 × 15 cm^2^ dose plane was exported to yield higher resolution.

### 2.5 IGRT performance evaluation

To assess synCT performance for IGRT, offline rigid registrations were performed between daily on‐board images and both simCT and synCT reference images. CBCTs were rigidly registered to synCT images using the Image Registration Workspace in Eclipse using six degrees of freedom (three translational and three rotational). The registration was then compared for equivalence to the corresponding CBCT‐simCT registrations to quantify in registration discrepancy. For patients with more than five CBCTs acquired during the treatment course, the first five CBCTs were chosen for evaluation. A total of 43 independent CBCT‐synCT and CBCT‐simCT registration pairs were compared. Two‐dimensional (2D) rigid registrations between on‐board kV images and DRR (n = 7) were completed using Elastix (University Medical Center Utrecht, Utrecht, Netherlands) via an in‐house MATLAB tool previously described.^17^, ^18^ Normalized mutual information (NMI) was used as the voxel‐based similarity metric^26^ to determine the translations in the registrations of both the anterior–posterior and lateral DRR images to their corresponding kV images.

### 2.6 Analysis for statistical comparisons

To assess the agreement between the simCT and synCT measurements, intraclass correlation coefficients (ICC)^27^ were computed for DVH metrics, CBCT‐CT, and kV‐DRR registration. To account for the correlations among multiple measurements from the same patient (e.g., primary and boost plan and multiple CBCT‐CT registrations), generalized estimating equations (GEE) methods were used to compare simCT and synCT agreement for the outcomes of DVH metrics and CBCT‐CT registrations. The standard errors address the correlations among multiple measurements from the same patient. Additionally, paired t‐tests were done to compare the simCT and synCT outcomes of DVH metrics, kV‐DRR, and CBCT‐CT registration. Differences with the p value less than 0.05 considered significant. All analyses were done using SAS version 9.4 (Cary, NC).

## 3 RESULTS

### 3.1 Dosimetric performance

#### 3.1.1 DVH metrics and plan quality

**Fig. 1:**
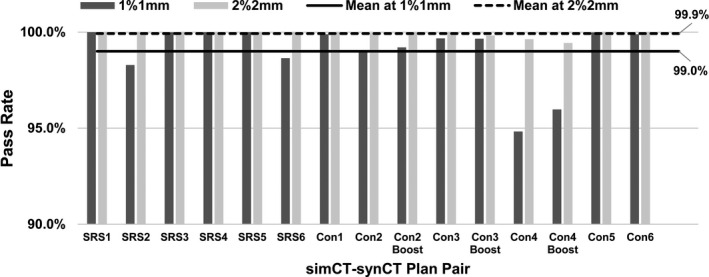
Gamma passing rates at 1%/1 mm and 2%/2 mm comparing dose distributions of synthetic CT (synCT) plan and of simulation CT (simCT) plan. *Abbreviations*: SRS = stereotactic radiosurgery; Con = primary plan of conventional brain radiotherapy; Con Boost = boost plan of conventional brain radiotherapy.

Table 1 summarizes key DVH metric results. Among 15 tested plan pairs, the mean difference (MD) was ≤0.10 ± 0.04 Gy for the target D_95%_ and ≤0.13 ± 0.04 Gy for OARs. While some statistically significant deviations were observed, the overall differences were deemed to not be clinically significant (i.e., low dose difference (<0.05 Gy)). The ICCs for evaluated DVH metrics were above 0.99, indicating excellent agreement between simCT and synCT plans. Across the entire cohort, close concordance in the GI for the synCT plans (3.88 ± 1.77, Range: 2.35 to 9.74) as compared to that of the simCT plans (3.76 ± 1.69, Range: 2.26 to 9.40) was observed. The maximum GIs of 9.74 and 9.40 for the synCT and simCT plans, respectively, occurred for a patient who had a simultaneous integrated boost with two separate target volumes treated with fractionated SRS to 32 Gy (Case SRS3 in Fig. 1). Similarly, the average CI was 1.12 ± 0.40 (Range: 0.49 to 2.26) and 1.14 ± 0.40 (Range: 0.59 to 2.33) for synCT and simCT plans for the entire cohort, respectively. No significant differences were observed in GI and CI between synCT plans and simCT plans (*P* > 0.05).

**Table 1:** Differences in dosimetric endpoints for the planning target volume (PTV) and organs at risk among 15 simCT/synCT plan pairs in 12 unique patients. Mean difference (MD) and standard error (SE) reported.

Dose Difference (Gy)	MD ± SE	[Min, Max]	*P*‐value
PTV			
D_95%_	0.10 ± 0.04	[−0.15, 0.35]	0.010*
D_99%_	0.05 ± 0.04	[−0.19, 0.29]	0.165
D_0.035 cc_	0.05 ± 0.04	[−0.30, 0.49]	0.277
Brainstem			
D_0.035cc_	0.13 ± 0.04	[−0.01, 0.43]	0.001*
D_0.5 cc_	0.09 ± 0.03	[0.00, 0.35]	0.001*
Chiasm			
D_0.035cc_	0.08 ± 0.03	[−0.01, 0.34]	0.003*
D_0.2 cc_	0.00 ± 0.08	[−1.06, 0.35]	0.966
Left Optic Nerve			
D_0.035 cc_	0.09 ± 0.04	[−0.01, 0.41]	0.007*
D_0.2 cc_	0.06 ± 0.03	[−0.13, 0.36]	0.020*
Right Optic Nerve			
D_0.035 cc_	0.09 ± 0.05	[−0.01, 0.79]	0.070
D_0.2 cc_	0.06 ± 0.03	[0.00, 0.40]	0.010*

* significant at *P* < 0.05.

#### 3.1.2 Gamma analysis

When comparing the synCT and simCT doses through an axial plane at isocenter, the gamma passing rates (γ < 1.0) averaged over 15 plans were 99.9 ± 0.2% (range, 99.4%–100%) at 2%/2 mm and 99.0 ± 1.5% (range, 94.8%–100%) at 1%/1 mm, as summarized in Fig. 1. Gamma analysis at 2%/2 mm criteria revealed similar passing rates between the SRS and conventional cases (100 ± 0.0% and 99.9 ± 0.2%, respectively). For 1%/1mm criteria, SRS yielded 99.5 ± 0.7% and conventional 98.7 ± 1.8% passing rates. The lowest gamma passing rates for the population occurred for conventional case 4 (99.6% at 2%/2mm and 94.8% at 1%/1mm) with similar performance for the corresponding boost plan (Con4 Boost), as shown in Fig. 1.

### 3.2 IGRT performance

The MDs between the CBCT‐synCT/simCT registrations and kV‐synCT DRR/simCT DRR registrations are summarized in Table 2. The population averaged MD in CBCT‐synCT registrations were <0.2 mm and 0.1 degree different from simCT‐based registrations. The largest differences were observed for synCT images associated with Con4 and Con4 Boost, with a maximum registration difference of 2.3 mm in S/I direction as compared simCT‐based registration. The MDs between kV‐synCT DRR/simCT DRR registration pairs were within 0.5 mm with no statistically significant differences observed (*P* > 0.05). The largest difference was again noticed in registrations related to synCT associated with Con4 and Con4 Boost: with registration difference of −1.62, −1.48, and 0.73 mm in R/L, A/P, and S/Is/i directions respectively.

**Table 2:** Differences of volumetric cone beam computed tomography (CBCT‐synCT/simCT, 43 observations in 12 subjects) and planar (kilovoltage (kV)‐synCT DRR/simCT DRR, 7 observations in 7 subjects) image registrations for image‐guided radiation therapy evaluation.

Registration	Translational Difference (mm)			Rotational Difference(°)		
CBCT‐CT	R/L	A/P	S/I	Pitch	Yaw	Roll
MD ± SE [Min, Max]	−0.04 ± 0.06 [−0.40,0.70]	−0.10 ± 0.06 [−1.10, 0.30]	−0.13 ± 0.16 [−2.30,1.60]	0.01 ± 0.04 [−0.40, 0.70]	0.05 ± 0.03 [−0.50, 0.50]	0.04 ± 0.03 [−0.40, 0.50]
P‐value	0.47	0.06	0.39	0.69	0.15	0.21

kV‐DRR	R/L	A/P	S/I	Transverse Plane	Sagittal Plane
MD ± SE [Min, Max]	−0.36 ± 0.30 [−1.62,0.91]	−0.20 ± 0.24 [−1.48,0.66]	−0.32 ± 0.17 [−0.11,0.88]	0.000 ± 0.002 [−0.004, 0.002]	0.000 ± 0.003 [−0.004, 0.006]
P‐value	0.28	0.45	0.11	0.78	0.77

Abbreviations: A/P, Anterior/Posterior; MD, mean difference; R/L, Right/Left; S/I, Superior/Inferior; SE, standard error.

### 3.3 Case studies

**Fig. 2:**
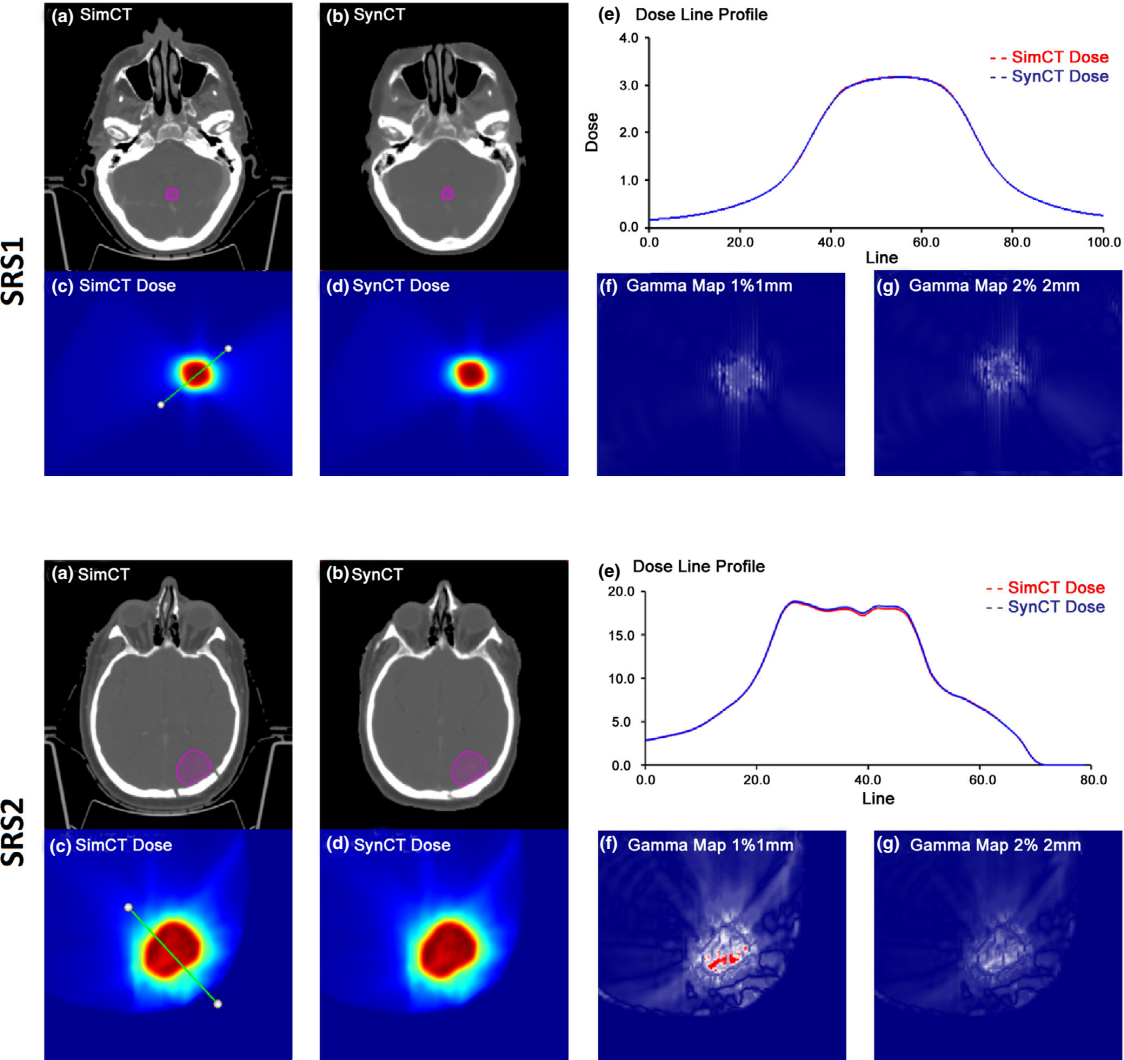
Two representative stereotactic radiosurgery (SRS) cases: SRS1 and SRS2. (a) and (b) demonstrate axial views of the simulation (simCT) and synthetic (synCT) at isocenter with the planning target volume (PTV) delineated; (c) and (d) show the corresponding dose distributions at the same axial plane; (e) illustrates the dose profiles along the line drawn on (c) through the PTV; (f) and (g) display the gamma map analyzed at 1%/1 mm and 2%/2 mm criteria, respectively.

Figure 2 summarizes dosimetric results for a typical SRS case (SRS1) and the case with the worst gamma results (SRS2). SRS1 presented with a small target (0.91 cc) seated in the middle of the brain. Note that the synCT maintained the same fine anatomical details as the simCT with the presence of air cavities and bone. Figures 2(c) and 2(d) demonstrate excellent dosimetric agreement in the target region between dose calculations as shown by the dose profiles from the synCT and simCT. The gamma criteria passing rates were 100% at both gamma criteria as seen in Figs. 2(e)–2(g). As a comparison, SRS2 had a larger metastatic lesion (17.2 cc) situated in close proximity to the skull affected by a region of surgical resection. Despite the synCT not representing the entire discontinuity in the skull, Fig. 2, part (e), for SRS2 showed overall good agreement between simCT and synCT dose profiles. As the worst‐case scenario in SRS cases, SRS2 achieved 98.3% and 100% gamma passing rates at 1%/1 mm and 2%/2 mm, respectively. IGRT evaluation yielded results similar to the population mean for both SRS1 and SRS2 cases.

**Fig. 3:**
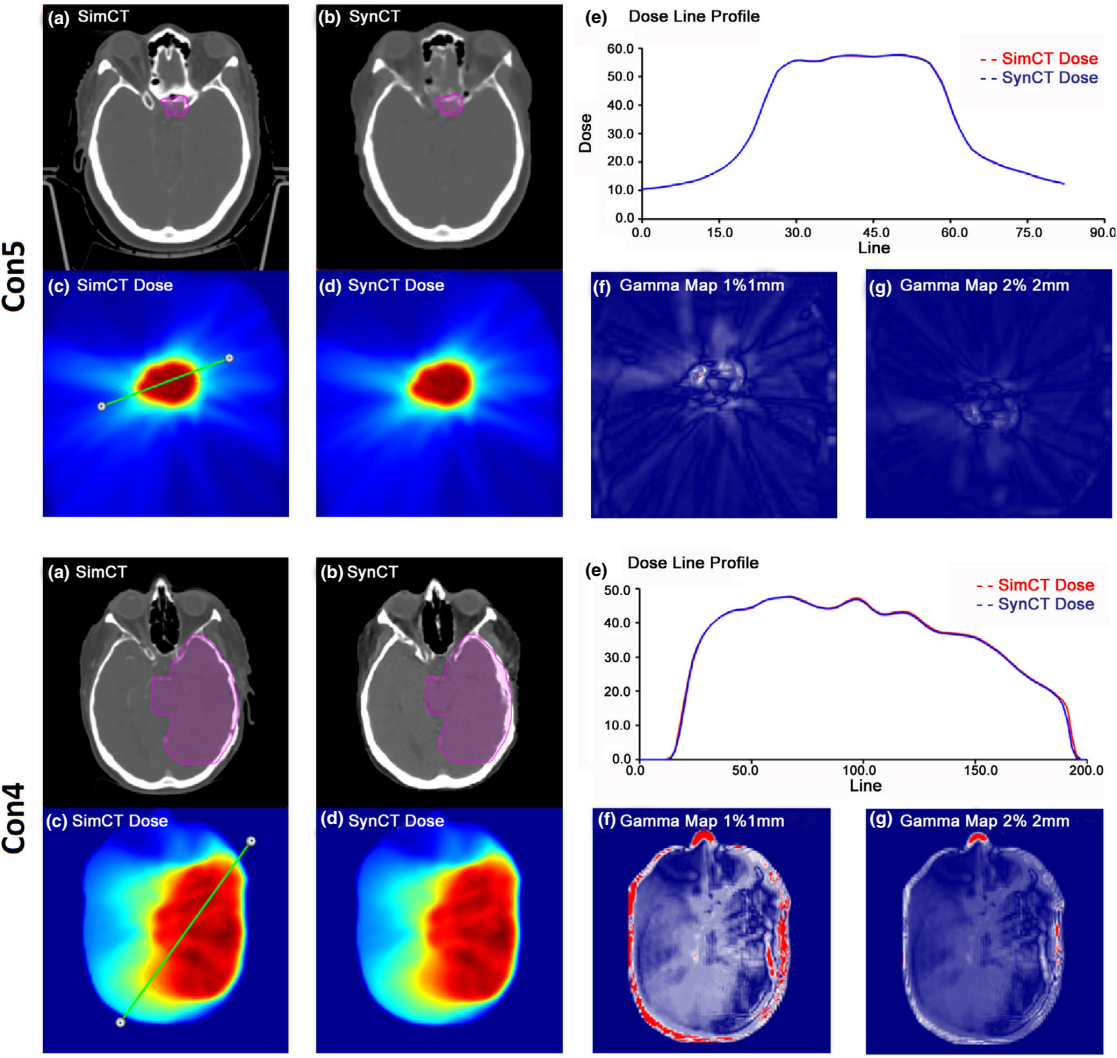
A typical conventional case (Con5) and the worst‐case scenario (Con4). (a) and (b) demonstrate axial views of the simulation (simCT) and synthetic (synCT) at isocenter with the planning target volume (PTV) delineated; (c) and (d) show the corresponding dose distributions at the same axial plane; (e) illustrates the dose profiles along the line drawn on (c) through the PTV; (f) and (g) display the gamma map analyzed at 1%/1 mm and 2%/2 mm criteria, respectively.

Figure 3 summarizes key results for the conventional radiation therapy cohort for a typical patient and a patient with lower dosimetric agreement between synCT and simCT. As shown in Fig. 3, Con5 consisted of a patient with recurrent cerebral meninges seated in the pituitary fossa abutting the anterior clinoid and treated to a total dose of 54 Gy using three DCAs. Although the synCT predicted decreased density in the bony region abutting the lesion (i.e., the tuberculum sellae), the dosimetric comparison revealed excellent agreement between synCT and simCT plans, as shown in the dose color wash and line profiles with 100% gamma passing rates at both 1%/1 mm and 2%/2 mm criteria.

**Fig. 4:**
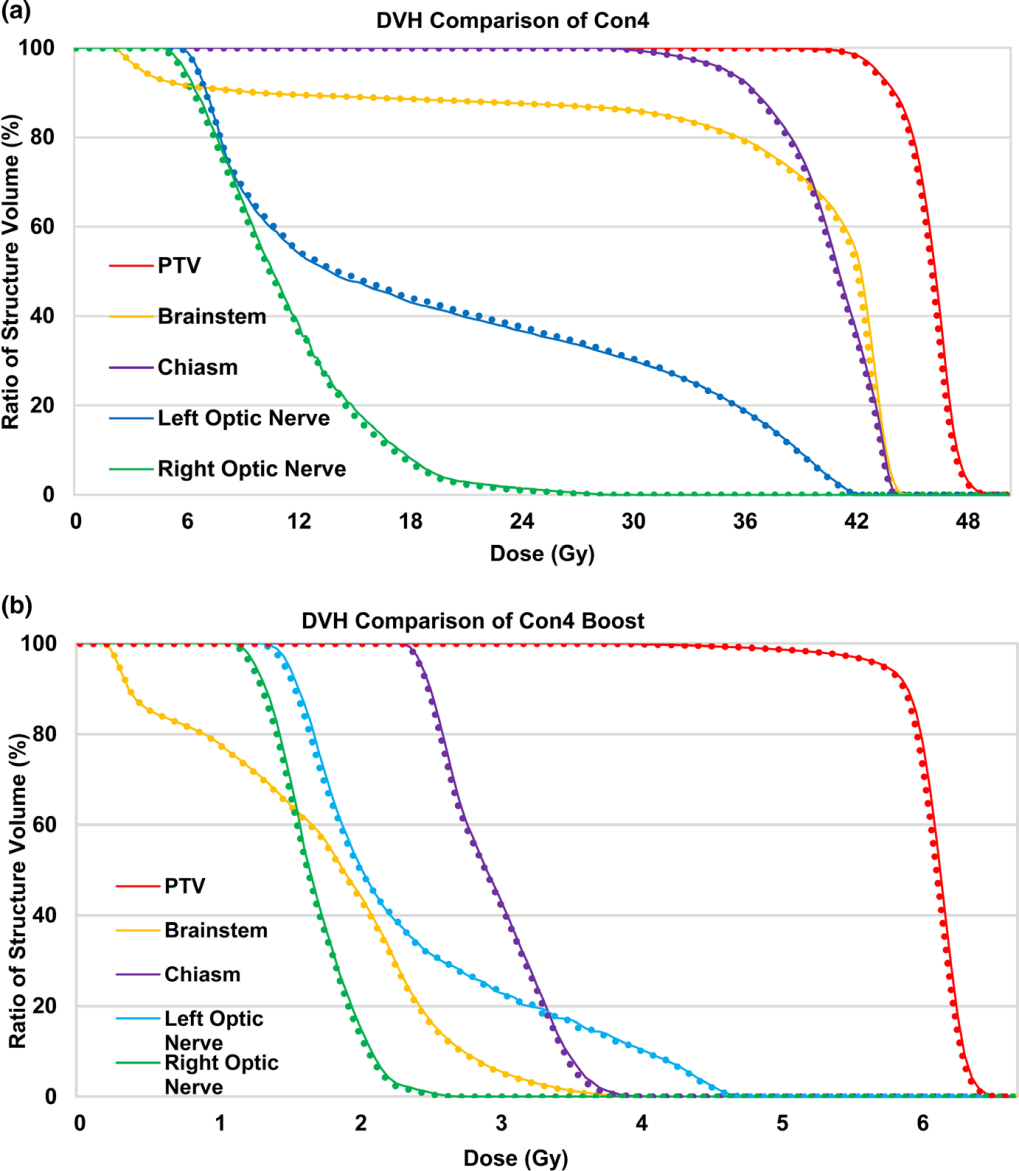
Dose volume histogram (DVH) comparison for conventional brain case 4 (Con4) that yielded the lowest gamma passing rates at isocenter. Nevertheless, minimal differences in the DVHs for both the primary (a) and boost (b) plans were observed. The solid curve represents simulation CT plan and the dotted curve represents synthetic CT plan.

By contrast, Con4 had a larger tumor (372.3 cc) that was situated in close proximity to the skull and had undergone a surgical resection. Compared with the corresponding simCT, the synCT showed an increased skull thickness, especially near the tumor volume. Nevertheless, the dose profile indicated good agreement across the target. Figures 3(f) and 3(g) for Con4 showed that the regions failing gamma criteria were along the periphery of the head and near the skull region that was impacted by surgical resection with gamma passing rates of 94.8% and 99.6% at 1%/1 mm and 2%/2 mm, respectively. For the same patient (Con4), the boost plan using the same synCT image yielded gamma passing rates of 96.0% at 1%/1 mm and 99.4% at 2%/2 mm. Despite the lowest gamma passing rates observed in this case, Fig. 4 illustrates that the DVHs were in good agreement for both Con4 (46 Gy in 2 Gy/fx) and Con4 Boost (14 Gy in 2 Gy/fx). Minimal differences between simCT and synCT DVH curves were also reflected by dose differences for clinical DVH metrics (<0.3 Gy for targets (D_95%_, D_99%,_ and D_0.035 cc_) and brainstem (D_0.5cc_ and D_0.035 cc_), and <0.6 Gy for optic pathways (D_0.2cc_ and D_0.035 cc_)). IGRT evaluation of this case showed that the registration differences were larger than the population average values. The CBCT‐simCT/synCT comparison revealed translational differences of 0, −0.34, and −1.34 mm in the right/left (R/L), anterior/posterior (A/P), and superior/inferior (S/I) directions, and rotational differences of −0.14, 0.14, and −0.08 degrees for pitch, yaw, and roll, respectively. This synCT also yielded the largest kV‐DRR registration deviation among all tested image pairs, with translational differences of −1.62, −1.48, and 0.73 mm at R/L, A/P, and S/I directions, respectively.

## 4 Discussion

This work sought to evaluate the dosimetric accuracy and IGRT performance of GAN‐synCTs generated for the brain for different clinical scenarios. Overall, results showed excellent agreement between synCT and simCT with DVH MEs <0.2 Gy. While 7 of 11 DVH metrics had statistically significant differences for the population, differences were not considered clinically significant due to the low dose difference (<0.05 Gy) when compared to prescription dose of at least 18 Gy. Generally speaking, excellent agreement in dose planes at the isocenter was observed between datasets (mean gamma passing rates were 99.9% and 99.0% at 2%/2 mm and 1%/1 mm, respectively), which surpassed the typical clinical criteria of 95% at 3%/3 mm.^28^ Our GAN‐synCT showed comparable results for accurate dose calculation to previously reported work by our group. Zheng *et al*. reported a mean gamma passing rate of 99.4% at 2%/2 mm for brain synCTs generated using a hybrid magnitude and phase MRI processing pipeline that required several input images for synCT generation.^14^ Another study evaluating brain synCT generated using dilated CNNs reported less than 1.5% difference in target dose as compared to the corresponding CT and yielded a mean gamma passing rate of 98.8% at 1%/1 mm.^29^ Wang *et al*.^15^ showed that brain synCTs generated from a path‐based random forest method in 14 brain SRS cases achieved less than 0.6% dose difference in target DVH metrics and a 99% average gamma passing rate at 3%/3 mm. Our work outperformed a more recent investigation on GAN‐based brain synCT that reported less than 1% dose difference for targets and OARs and gamma passing rates of 98.7% and 93.6% at 2%/2mm and 1%/1mm, respectively.^29^ Our work adds to the literature by also considering IGRT performance for our cohort, as well as quantifying the dosimetric impact.

IGRT evaluation showed that differences between synCT‐based and simCT‐based registrations were minimal. The MDs between volumetric registration pairs were <0.2 mm and <0.1°. The largest discrepancy occurred for CBCT‐guided conventional cases in the S/I direction, likely due to the 2.5 mm slice thickness that may lead to increased registration uncertainties. Similarly, Gupta *et al*.^30^ reported MEs of −0.1, −0.1, and −0.2 mm in the A/P, R/L, and S/I directions, respectively, for synCTs generated using a deep learning U‐Net architecture trained on sagittal T1‐weighted MRI datasets. For orthogonal planar registrations, GAN‐synCT achieved <0.4 mm and 0.01 degree differences as compared to simCT which were comparable to those observed by Price *et al*. evaluating synCTs generated through a voxel‐based weighted summation method (0.4 ± 0.5 mm, 0.0 ± 0.5 mm, and 0.1 ± 0.3 mm in S/I, L/R, and A/P directions, respectively).^17^

One limitation of present work is the small cohort of patient (12 patients, 15 plans) from a single institution and MRI scanner. Nevertheless, a variety of patient conditions and plans, including state of the art DCA and SRS, were considered to test the GAN‐synCT performance over a range of settings. GAN‐synCT maintained most of the details in simCT although some differences were noted in regions of the skull that contained underestimates of the discontinuities along air‐tissue interfaces. The worst performing case in the cohort highlighted in Figs. 3(f) and 3(g) for Con4 suggested that the regions failing gamma criteria occurred near the periphery of the head. Dose distributions along peripheral regions are susceptible to partial volume effect due to different resolutions of simCT and synCT. Other potential sources of uncertainty are the presence of immobilization devices in simCT but not in the MR‐SIM acquisition used to generate synCTs as well as any residual coregistration uncertainties used in the dosimetric evaluation. Previous studies reported similar challenges in accurate synCT generation at air‐bone and air‐tissue interfaces.^6^, ^31^ Han^32^ generated synCTs using deep CNNs and observed larger errors at the bone and air boundary due to high intensity gradients and imperfect alignment between MR and simCT. Paradis et al.^31^ showed larger dose discrepancy caused by differences in the regions of close proximity of air cavities between simCT and synCT generated from multiple MR images using probabilistic voxel classification. Although in this study, the resulting dose differences were found to be clinically insignificant, an ideal synCT generation would be more robust and address these discontinuities.

Another source of uncertainty was observed near skull regions that are impacted by surgical resection where the synCT tended to overestimate the skull thickness in the surgical cavity region as compared to simCT such as in Fig. 3 (Con4). This led to increased discrepancies in volumetric IGRT performance with a maximum registration difference of 2.3 mm in S/I direction as compared simCT‐based registration. While potential dose disagreement may be possible due to this performance if the tumor was in close proximity to this region, no clinically significant dose deviations in plan quality or IGRT performance were observed in this study. However, this suggests that caution needs to be exercised in patients with atypical anatomy that may exhibit larger errors than this cohort. These discrepancies can be addressed by visual evaluation during the training phase of the deep learning algorithm and by integrating additional atypical anatomy into the training set. An area of emerging interest is developing spatial attention‐guided GAN^33^ to minimize the differences between simCT and synCT in areas of increased discrepancy that may offer additional improvements in postsurgical settings. Nevertheless, GAN‐synCT demonstrated high accuracy in dose calculation and image registration. Confirming this work in a larger cohort using different MRI field strengths is warranted. Of note is that not all patients will be candidates for MR‐only treatment planning. Future studies can fully evaluate potential uncertainties and failure modes via effects analysis as we have previously conducted in pelvis MR‐only planning.^34^

## 5 Conclusion

GAN‐synCT was evaluated in terms of dosimetric and IGRT accuracy for brain radiotherapy. Results showed comparable performance of synCT under multiple clinical settings as compared to standard of care simCT. This work illustrates the feasibility of clinical implementation of GAN‐synCT in MR‐only radiotherapy for brain cancer.

## Conflict of Interest

Dr. Glide‐Hurst reports research agreements with Modus Medical, ViewRay Inc., and Philips Healthcare and participation on the Philips Advisory Board unrelated to the current work.

## Author contribution

Xiaoning Liu, M.S.: Treatment planning generation, data analysis, perform repeat registrations, manuscript preparation, and figure preparation. Hajar Emami‐Gohari, B.S.: Development of deep learning pipeline, synthetic CT generation, and manuscript review. Siamak P. Nejad‐Davarani, Ph.D.: Image preprocessing, coregistration of synthetic CT and CT datasets needed for treatment planning, and manuscript review. Eric Morris, Ph.D.: Image registration tool in MATLAB, automated analysis generation, figure preparation, and manuscript review. Lonni Schultz, Ph.D.: Statistical analysis. Ming Dong, Ph.D., Assistance with deep learning optimization for synthetic CT, and manuscript review. Carri K. Glide‐Hurst, PhD, PI of IRB study, PI of grant supporting research, study conception, analysis, and manuscript and figure preparation.
